# Sclerosing mesenteritis in a 5-year-old Chinese boy: a case report

**DOI:** 10.1186/s12887-017-0931-8

**Published:** 2017-08-01

**Authors:** Cui-ping Liang, Min Yang, Pei-Yu Chen, Lan-Lan Geng, Ding-You Li, Si-Tang Gong

**Affiliations:** 1 Department of Gastroenterology, Guangzhou Women and Children’s Medical Center, 9 Jinsui Road, Guangzhou, 510623 China; 2 Department of Pediatrics, Division of Gastroenterology, University of Missouri-Kansas City, Children’s Mercy Hospital, Kansas City, MO USA

**Keywords:** Sclerosing mesenteritis, Intestinal obstruction, Child

## Abstract

**Background:**

Sclerosing mesenteritis is a rare fibroinflammatory disorder of unknown etiology that primarily affects the mesentery of the small intestine during late adult life. Only about twenty pediatric cases have been reported to date, but none has been reported in Chinese children.

**Case presentation:**

A 5-year-old Chinese male presented with a 4-week history of recurrent bloating, abdominal pain, anorexia and vomiting. On admission, physical examination showed a severely distended abdomen. Biochemical investigations showed a slightly increased C-reactive protein, and normal serum levels of electrolytes and erythrocyte sedimentation rate. An abdominal film showed small intestine obstruction and massive ascites. An exploratory laparotomy revealed widespread inflammatory fibrotic adhesions between the bowel and the abdominal wall, thickening of the small bowel and massive ascites. During a prolonged hospital course, a 2nd surgery (4 months after 1st exploratory laparotomy) was performed in order to close the ileostomy and revealed that the bowel was still severely edematous, with very tight adhesions between the bowel and the abdominal wall. Histopathological examination of excised mesentery and nodules showed chronic inflammatory cell infiltration, fat necrosis and fibrosis. A diagnosis of sclerosing mesenteritis was finally established. Prednisolone at 2 mg/kg was started and he experienced rapid clinical improvement in 4 weeks.

**Conclusions:**

Sclerosing mesenteritis is extremely rare in children and often misdiagnosed due to its nonspecific clinical manifestation. It is important to be aware of sclerosing mesenteritis when evaluating a child with intractable abdominal pain, bloating, intestinal obstruction and massive ascites.

## Background

Sclerosing mesenteritis is a rare non-neoplastic disorder of unknown etiology that primarily affects the mesentery of the small intestine with chronic fibrosing inflammation during late adult life [1–3]. Only about twenty pediatric cases have been reported to date [2], but none has been reported in Chinese children. It can be an acute or insidious onset and lacks specific clinical manifestations. Abdominal pain and mass are the main symptoms. Here we report the first case of 5-year-old Chinese boy with sclerosing mesenteritis who presented with intractable abdominal pain, bloating, intestinal obstruction and massive ascites.

## Case presentation

**Fig. 1:**
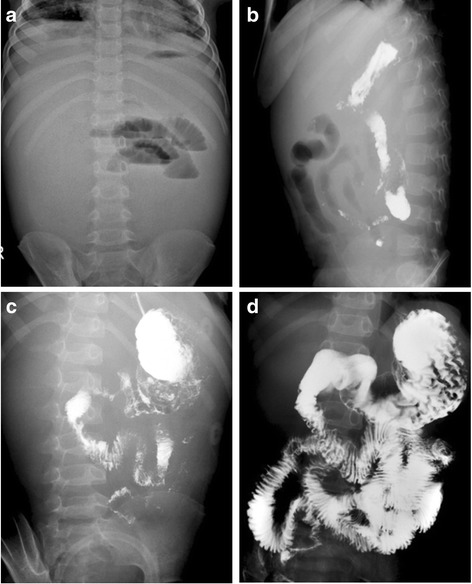
Imaging studies. **a** The abdominal film showed ileus and massive ascites; **b** The initial barium study showed partial jejunum obstruction and slow transit with only a small amount of barium reaching to the distal jejunum; **c** The 2nd barium study showed slow bowel peristalsis, and delayed transit in the duodenum and jejunum; **d** The 3rd barium study showed mildly delayed gastric emptying and delayed transit in jejunum

A 5-year-old Chinese boy presented with a 4-week history of recurrent bloating, abdominal pain, anorexia and vomiting. There was no past or family history of any IgG4 and/or auto-immune related diseases. On admission, physical examination showed a moderately distressed patient with a temperature of 36.2 °C, a respiratory rate of 23 breaths/min, a blood pressure of 92/63 mmHg and a pulse rate of 102 beats/min. Abdominal examination revealed a severely distended abdomen with massive ascites. There was positive shifting dullness and diminished bowel sounds, but no abdominal wall rigidity. A complete blood count revealed a white blood cell count of 10.2 × 10^9^/L with 64% neutrophils, a hemoglobin level of 108 g/L and hematocrit of 35%. Biochemical investigations showed significantly increased serum levels of IgE (280 IU/ml; normal reference 0 IU/ml to 30 IU/ml) and C-reactive protein (11 mg/L; normal reference < 0.5 mg/L). Liver transaminases, albumin, total bilirubin, alkaline phosphatase, glucose, erythrocyte sedimentation rate, electrolytes and renal function tests were all within the normal ranges. Both stool culture and parasite tests were negative. An abdominal CT scan revealed bowel wall thickening on the right side and massive ascites. A positron emission tomography was performed to exclude any malignancies and showed a large soft tissue density around the pancreas, with inflammatory lesions and mild metabolic activity. There was widespread bowel edema. A diagnostic paracentesis revealed slightly yellow-colored fluid containing white blood cells (1000 × 10^9^/L). Rivalta test of the ascites was positive for exudate and there was no tubercle bacillus or malignancy. An abdominal film showed intestinal obstruction (Fig. 1a). An initial barium study suggested an incomplete jejunal obstruction with delayed transit and only a small amount of barium reaching the distal jejunum (Fig. 1b).

An exploratory laparotomy was performed. There were widespread dilatation, edema and thickening of the small bowel, with massive clear slightly-yellowish ascites of about 1300 ml. Small intestine mesentery and ascending colon were also thickened, with multiple yellowish nodules. There were extensive intraperitoneal adhesions. No evidence of obstructive pathology was identified. The disease was centered more on the mesentery. With concern for malignancy, nearly 60 cm of bowel including the terminal ileum, ileocecal valve, part of ascending colon and their mesentery, and part of omentum, were resected. An end ileostomy was created.

**Fig. 2:**
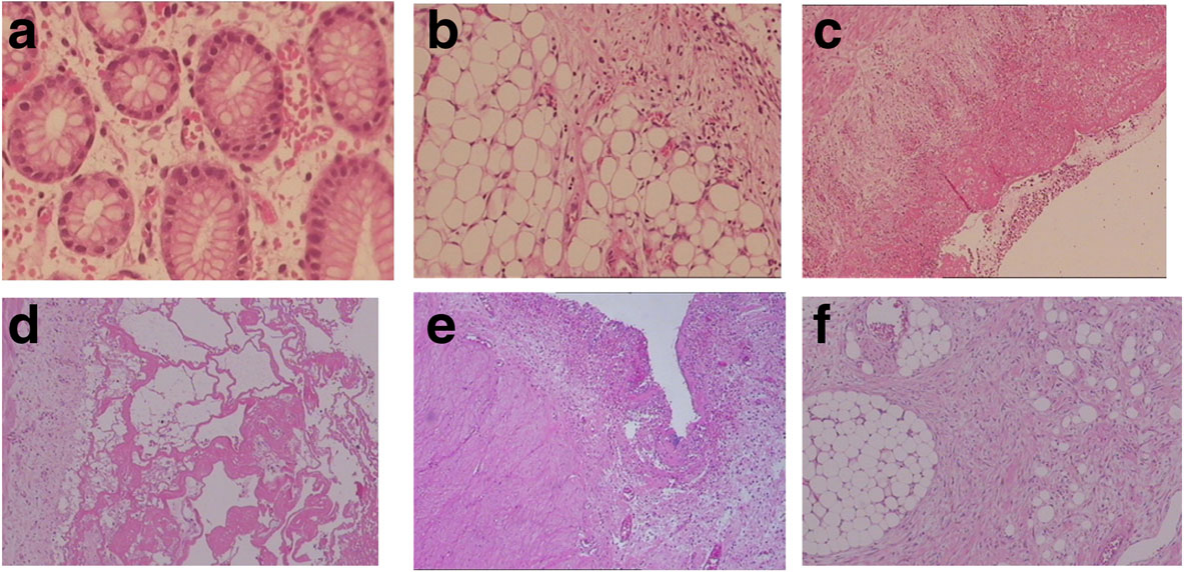
First histopathological examination showed (1) eosinophilic infiltration (5-30 /HPF) in the lamina propria (2A); (2) fibrous connective tissue hyperplasia in omentum, lymphocytic infiltration and adipose tissue (2B); and (3) mesentery tissue with inflammatory cell infiltration, fat necrosis, fibroblastic proliferation and fibrosis (1C). The 2nd histopathological examination (4 months after initial surgery) showed inflammatory cell infiltration and fibrinoid necrosis in serosal surface (2D; mesentery tissue with inflammatory cell infiltration, fibroblasts proliferation (2E) and fat necrosis (2F)

Histopathological examination showed significant thickened serosa and eosinophilic infiltration (5-30/high power field) in the lamina propria. Mesentery and nodules had inflammatory cell infiltration, fat necrosis, fibroblast proliferation and fibrosis. There was omental fibrous connective tissue hyperplasia with infiltration of lymphocytes and adipose tissue (Fig. 2 a-c).

Post-surgically, the patient was treated with parenteral nutrition, intravenous fluids, gastrointestinal decompression, and pro-motility agents such as mosapride and erythromycin. However, severe bloating persisted and more than 1000 ml of green-yellowish fluids was drained from the decompression tube and 200 ml from the ileostomy daily. With suspicion of eosinophilic enteritis based on pathology report, he was treated with intravenous dexamethasone at 0.2 mg/kg/d, which was gradually decreased to 0.1 mg/kg/day over a 2-week period and stopped after 4 weeks. His abdominal pain decreased and ascites resolved, with a gradual improvement in enteral nutrition intake. While the decompression tube drainage was decreased, the ileostomy drainage increased to more than 1000 ml daily. The second and third barium studies showed some degree of gastrointestinal motility improvement (Fig. 1c and d). Subsequently at 4 months after the initial exploratory laparotomy, a 2nd surgery was performed in order to close the ileostomy and revealed that the bowel was still severely edematous, with very tight adhesions between the bowel and the abdominal wall. Ileostomy closure couldn’t be performed. Tissues near the ileostomy, including bowel and mesentery, were resected and sent for pathological examination, which showed chronic inflammatory cell infiltration, fat necrosis and fibrosis (Fig. 2 d-f). Based on pathological features and clinical presentations, a diagnosis of sclerosing mesenteritis was finally established. Prednisolone at 2 mg/kg was started and he experienced rapid clinical improvement in 4 weeks and was discharged from the hospital. After 8 weeks of treatment, prednisolone was slowly weaned and stopped after 24 weeks. At 8-month after hospital discharge, he was asymptomatic with adequate weight gain. A final surgery (15 months after the initial exploratory laparotomy) was performed to close the ileoostomy and there was no edema but he was noted to have diffusely enterocolonic adhesions. The boy remained asymptomatic at a 6-month post-operation clinic visit.

## Discussion

Sclerosing mesenteritis is a rare chronic fibrosing inflammatory disease of ambiguous pathogenesis primarily diagnosed in late adult life while pediatric cases are very uncommon because children have mesenteric fat than adults [1–3]. It is a benign disorder mainly involving the small-bowel mesentery; however, mesocolon, peripancreatic region, omentum, pelvis or retroperitoneum may be also involved. Both mesenteric panniculitis and mesenteric lipodystrophy are considered to be histological variants of sclerosing mesenteritis [4]. Viswanathan and Murray reported one case and summarized another 16 pediatric cases from the literature and found that the average age at diagnosis in children was 6.5 years [2]. In our case, the lesion was extensive and involved small intestine, ascending mesocolon, omentum and peritoneum, leading to intractable bowel obstruction. Clinical symptoms are usually non-specific, including abdominal pain, anorexia, fatigue, weight loss, fever, abdominal mass, ascites, pericardial effusion, diarrhea and constipation [1–3]. Laboratory examinations including C-reactive protein, erythrocyte sedimentation rate, complete blood count, and biochemistry are also non-specific, which renders a diagnostic challenge for clinicians. Abdominal CT or ultrasonography may play an important role in diagnostic evaluation and the most common finding is a soft tissue mass at the root of small bowel mesentery [5]. In the present case, massive peritoneal cavity effusion resulted in a negative finding on CT scan. A definite diagnosis is dependent on histopathology, which usually reveals fat necrosis, chronic inflammation and fibrosis but can be variable from case to case. These histological variants may reflect the state of a chronic inflammatory process; necrosis in mesenteric fat may represent disease progression and eventually progress to fibrosis which leads to retractile mesenteritis [6]. In the present case, infiltration of chronic inflammatory cells, fat necrosis and fibrosis were all observed in the histological findings from initial and subsequent surgical specimens, but due to lack of awareness of the disease, a diagnosis of sclerosing mesenteritis was not recognized at the first operation. Therefore, due to the nonspecific signs and symptoms of sclerosing mesenteritis, diagnosis is mainly made through a combination of histopathological and imaging (preferably abdominal CT) findings [7].

A recently defined subtype, IgG4-related sclerosing mesenteritis, has been reported to be closely related to IgG4-related disease and one such case was reported in children [8–10]. In our case, IgG4 serum subclass levels or tissue immunohistochemistry were not performed.

The optimal therapeutic strategy for sclerosing mesenteritis remains unclear. Some asymptomatic or mild clinical cases may resolve spontaneously without therapy. In some cases with intensive fibrosis leading to intractable bowel obstruction or perforation, surgical resection or ileostomy might be required [1–3]. Medications including corticosteroids, thalidomide, cyclophosphamide, colchicine, azathioprine and tamoxifen have been reported to induce remission of sclerosing mesenteritis [11–13]. Most of the previous reported pediatric cases were successfully treated with corticosteroids. Our patient was started on corticosteroids at 2 mg/kg after definitive diagnosis was made and had a clinical remission in 4 weeks. Due to the severity of intestinal inflammation, we decided to keep him on corticosteroids at 2 mg/kg for another 4 weeks and then slowly weaned off it over 6 months.

## Conclusions

We describe a very rare case of sclerosing mesenteritis in a Chinese boy. We failed to make a diagnosis at the first operation due to lack of awareness of the disease. Sclerosing mesenteritis should be considered as a differential diagnosis in patients with intractable bowel obstuction and serous cavity effusion. A definite diagnosis is dependent on histopathology. Judicious use of corticosteriods therapy can lead to clinical remission.
